# Evaluating Research Preparedness and Skill Gaps Among Osteopathic Medical Students Pursuing Surgical Specialties

**DOI:** 10.7759/cureus.99157

**Published:** 2025-12-13

**Authors:** Skylarr A Beerman, Brenton Stucki, Ryan Wong, Vincent S Alexander, Andrew Vogel, Matthew A Heard, Tyler J Wallen

**Affiliations:** 1 Department of Research, New York Institute of Technology College of Osteopathic Medicine at Arkansas State University, Jonesboro, USA; 2 Texas College of Osteopathic Medicine, University of North Texas Health Science Center, Forth Worth, USA; 3 Department of Medicine, Nova Southeastern University Dr. Kiran C. Patel College of Osteopathic Medicine, Fort Lauderdale, USA; 4 Department of Head and Neck Oncology, Alabama College of Osteopathic Medicine, Dothan, USA; 5 Department of Cardiothoracic Surgery, University of North Carolina at Chapel Hill, Chapel Hill, USA; 6 Department of General Surgery, East Tennessee State University Quillen College of Medicine, Johnson City, USA; 7 Department of Cardiovascular Surgery, Geisinger Commonwealth School of Medicine, Wilkes-Barre, USA

**Keywords:** mentorship, osteopathic medical students, research skills, residency applications, surgical education

## Abstract

Objectives

The objective of this study is to evaluate osteopathic medical students’ self-reported research experiences, skill levels, and professional goals. This study specifically aimed to assess how students perceive their readiness to engage in research, explore the barriers they face in translating motivation into scholarly productivity, and identify which research tasks students feel most and least confident performing. These findings aim to clarify the disconnect between strong research motivation and limited output and to inform future efforts to improve research engagement among osteopathic students pursuing surgical careers.

Methods

A national, cross-sectional survey was administered to osteopathic medical students interested in surgical specialties. The survey assessed demographic characteristics, prior research experience, confidence in research skills, and future research goals. Responses were stratified by confidence in research, and associations between prior research activity and confidence in specific research skills were analyzed using the Cochran-Armitage test for trend.

Results

Among 75 respondents, 86.7% had never coauthored a peer-reviewed publication, and 70.7% had never published as a first author. Despite this, students with prior poster presentations reported high confidence in designing and executing research (83.3%), preparing manuscripts (92.6%), presenting research (81.5%), and teaching research fundamentals (90.9%) (p < 0.05). Podium presentations were associated with high confidence in designing research (55.6%), preparing manuscripts (60.9%), presenting (51.9%), and teaching (54.5%). Coauthor publications were associated with confidence in designing research (69.4%), manuscript preparation (70.7%), and teaching (63.6%), while first-author publications were associated with confidence in the same domains (38.9%, 41.5%, and 42.4%, respectively). Quickshot presentations were associated only with manuscript preparation (41.5%) (p < 0.05).

Conclusions

Osteopathic medical students interested in surgical specialties report confidence and motivation to participate in research but lack prior research presentations and publications, as well as statistical and data analysis skills. These findings highlight the need for structured research support, mentorship, and training in research methodology and analysis. To address these needs, national research program initiatives have been developed to provide osteopathic students with access to surgical research opportunities, mentorship, and skill-building resources. Such initiatives may help bridge the gap between student motivations and research productivity, improving equity and competitiveness in the residency selection process.

## Introduction

Research exposure plays an important role in medical education. Early involvement in research introduces students to the foundations of evidence-based clinical practice, teaches the fundamentals of study design, and builds familiarity with biostatistics, all skills that are essential throughout a physician’s career [1]. Additionally, research involvement may secondarily improve the competitiveness of a residency application. In recent years, research experience and productivity have become increasingly important during the medical school journey, especially for students pursuing competitive surgical specialties [2]. This trend has further intensified since the Comprehensive Osteopathic Medical Licensing Examination of the United States (COMLEX-USA) Level 1 and the United States Medical Licensing Examination (USMLE) Step 1 shifted to pass/fail scoring systems. This transition has led residency program directors to place greater emphasis on other aspects of residency applications, such as research involvement and outcomes, when evaluating applications for residency selection [3].

For instance, a 2021 national survey reported that 41% of residency program directors predicted research would play a larger role in interview decisions after the scoring transition [3]. The proportion of general surgery program director respondents emphasizing a “demonstrated involvement and interest in research” increased from 38% in 2020 [4] to 56% in 2024 [2]. Similar trends were observed in other competitive specialties, including neurological surgery, orthopedic surgery, and vascular surgery, underscoring the increased emphasis on research productivity, such as presentations and publications, in the post-numeric scoring era of COMLEX-USA Level 1 and USMLE Step 1.

Despite higher expectations, not all students have equitable access to meaningful research opportunities. According to the National Resident Matching Program (NRMP) data, matched osteopathic applicants in surgical fields typically report fewer research experiences and publications compared to their allopathic peers [5,6]. Prior studies have highlighted common barriers, such as difficulty finding mentors, uncertainty in initiating their own projects, and a lack of institutional support [7]. These challenges may prevent motivated and capable students from converting their interests into tangible research outcomes. Currently, there remains a limited understanding of how osteopathic medical students perceive their own research skills and preparedness to contribute to research projects.

To address this gap, a national survey of osteopathic medical students interested in surgical specialties was conducted. This study aimed to assess osteopathic medical students’ self-reported research experiences, skill levels, and professional goals. Specifically, the study aimed to (1) evaluate how students perceive their readiness to engage in research; (2) explore the barriers they face in translating motivation into tangible scholarly output; and (3) identify which research tasks students feel most and least confident performing. By clarifying this disconnect between high interest and low productivity, the study seeks to fill a gap in the literature and inform the development of initiatives that more effectively support research engagement among osteopathic students pursuing surgical careers.

## Materials and methods

Survey population

Eligible participants included osteopathic medical students enrolled at accredited colleges of osteopathic medicine in the United States who self-reported an interest in surgical specialties. Participation was voluntary. No formal sample size calculation was performed due to the exploratory nature of this study. Respondents were excluded if they did not meet these criteria or submitted incomplete survey responses. Only responses with complete data for all survey items were included in the final analysis. Partial responses were excluded, and no imputation was performed.

Survey instrument and recruitment

A cross-sectional survey titled the Research Skills and Goals Assessment Survey was developed to evaluate research experiences, self-assessed research skill levels, and future research goals among osteopathic medical students. The full survey is included in Appendix A. To ensure content validity, the survey was developed using findings from prior research demonstrating that osteopathic medical students have fewer research experiences and publications when applying for residency [5-7]. This approach helped confirm that the survey items were comprehensive and aligned with our hypothesis that osteopathic medical students encounter barriers that limit the progression of research interest into completed work. No formal pilot testing or pretesting was conducted. However, three senior investigators independently reviewed each survey item to ensure content and face validity and clarity for the study’s target population.

The survey was created using an online survey platform (Google Forms, Google LLC, Mountain View, CA). All students were shown the same set of questions, in a fixed order, without skip patterns or branching logic. The survey consisted of multiple-choice, Likert-scale (1 = strongly disagree to 5 = strongly agree), and short-answer items.

The survey was distributed using three methods: (1) monthly email dissemination from November 2024 through April 2025 through the American College of Osteopathic Surgeons - Medical Student Section (ACOS-MSS); (2) social media promotion on X (formerly Twitter), with posts published on January 20, 2025, and March 8, 2025; and (3) in-person at the 2025 ACOS-MSS Spring conference, held April 12-13, 2025. Flyers containing a QR code linked to the survey were distributed and displayed throughout the conference venue, and students were encouraged to participate voluntarily. While no formal stratification or randomization was performed, the study employed broad dissemination strategies to reach a geographically diverse and representative sample of osteopathic medical students.

Statistical analysis

Descriptive statistics were used to summarize categorical demographic variables. The Cochran-Armitage test for trend was used to assess linear associations between levels of prior research experience and ordinal Likert responses, identifying patterns in confidence and perceived skill. Five-point Likert responses were consolidated into three categories: low (1-2), medium (3), and high (4-5). This approach maintained the theoretical structure of the scale while reducing noise and accommodating a limited sample size. Research experience variables (e.g., podium, poster, and quickshot presentations; first- or coauthor publications) were dichotomized by participation (yes/no). Only complete responses were included. Missing data were excluded from analyses. All statistical testing was conducted using IBM SPSS Statistics for Windows, Version 30.0 (Released 2024; IBM Corp., Armonk, NY, USA), with p-values < 0.05 considered statistically significant.

## Results

A total of 75 participants completed the survey. Table 1 summarizes the demographic characteristics of the respondents. Most respondents were under 30 years old, with 44% aged 18-25 and 52% aged 26-30. The majority of respondents identified as White (62.7%), followed by Asian (32%). The majority identified as having a middle-income socioeconomic status (63.5%). Only a small proportion of participants reported having a surgical residency program at their home institution (n = 11, 14.7%). Most had prior research experience (n = 65, 86.7%), and a substantial majority were currently involved in research projects (n = 60, 80%). Familiarity with research methodologies was highest for case reports (n = 53, 71.7%), systematic reviews (n = 51, 68%), and basic science research (n = 46, 61.3%). Respondents had the lowest familiarity with clinical trials (n = 26, 34.7%) and meta-analyses (n = 31, 41.3%).

**Table 1:** Demographic characteristics of survey respondents

Variable	Response	N (%)
Sex	Male	43 (57.3%)
	Female	32 (42.7%)
Age	18-25	33 (44%)
	26-30	39 (52%)
	31-40	3 (4%)
Race	White	47 (62.7%)
	Black or African American	3 (4%)
	Asian	24 (32%)
	Other	3 (4%)
Military service	Yes	7 (9.3%)
	No	68 (90.7%)
Socioeconomic status	Below the poverty line	5 (6.7%)
	Low income	5 (6.7%)
	Middle income	49 (65.3%)
	High income	16 (21.3%)
Type of medical school	University/academic	27 (36%)
	Community/community-affiliated	34 (45.3%)
	Mix of academic and community	14 (18.7%)
Geographic location of the medical school	Pacific	1 (1.3%)
	Mountain	7 (9.3%)
	West North Central	13 (17.3%)
	West South Central	16 (21.3%)
	East North Central	2 (2.7%)
	East South Central	15 (20%)
	New England	2 (2.7%)
	Middle Atlantic	5 (6.7%)
	South Atlantic	14 (18.7%)

**Figure 1:**
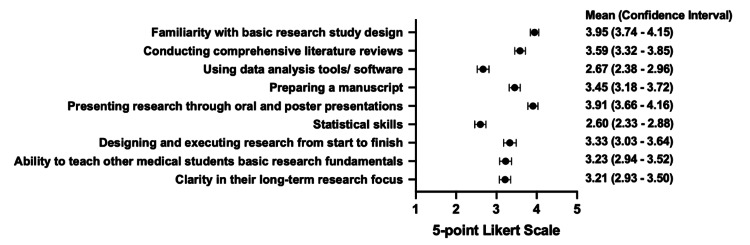
5-point Likert-scaled responses for self-perceived research competency variables (n = 75) Data points are shown as means ± standard error of the mean.

Figure 1 displays the mean Likert-scale responses for each self-perceived research competency. The highest-rated competencies included familiarity with basic research design (mean = 3.95), presenting research through oral or poster presentations (3.91), and conducting comprehensive literature reviews (3.59). The lowest-rated competencies were statistical skills (2.60) and proficiency in data analysis tools or software (2.67). Individuals had moderate clarity in their long-term research focus (3.21).

**Figure 2:**
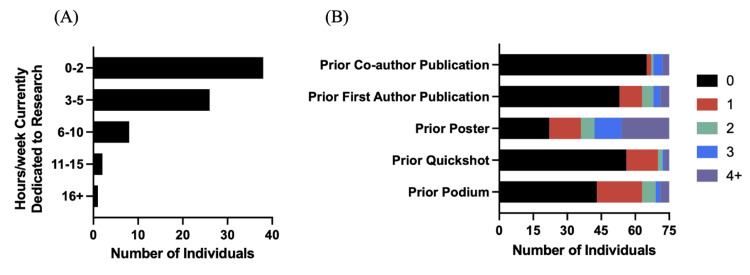
(A) Number of hours per week osteopathic medical students currently dedicate to research. (B) Prior scholarly output among osteopathic medical students.

Figure 2 illustrates participants’ prior scholarly output and current research involvement. Over half (50.7%) reported conducting less than two hours of research per week, while 34.7% reported spending three to five hours weekly (Figure 2A). Most had completed at least one poster presentation (n = 53, 70.7%). (Figure 2B) However, the majority had not authored a first-author publication (n = 53, 70.7%) or a coauthor publication (n = 65, 86.7%).

Tables 2-5 present the associations between prior scholarly output and self-perceived confidence in specific research competencies. Prior research experiences were not significantly associated with confidence in familiarity with study design principles, literature reviews, data analysis tools, or statistical skills. However, students with prior poster presentations reported significantly higher confidence in designing and executing research projects (83.3%, p = 0.005), preparing manuscripts (92.6%, p < 0.001), presenting research (81.5%, p < 0.001), and teaching research fundamentals (90.9%, p < 0.001). Quickshot presentations were significantly associated with confidence in preparing manuscripts (41.5%, p < 0.001) and teaching research (47.8%, p = 0.040). Podium presentation experience was associated with confidence in manuscript preparation (60.9%, p < 0.001), presenting research (51.9%, p = 0.005), designing and executing research (55.6%, p = 0.019), and teaching (54.5%, p = 0.006). Students with coauthor publications reported significantly higher confidence in designing and executing research (69.4%, p = 0.019), manuscript preparation (70.7%, p = 0.008), and teaching research (63.6%, p = 0.033). First-author publications were also associated with increased confidence in designing and executing research (38.9%, p = 0.024), manuscript preparation (41.5%, p = 0.009), presenting research (46.4%, p = 0.025), and teaching (42.4%, p = 0.003).

**Table 2:** Associations between prior research activities and self-perceived research familiarity and design among osteopathic medical students interested in surgery Numbers in each cell represent the count of respondents (n) within a given confidence category who reported the corresponding prior research activity. Percentages in parentheses reflect the proportion of individuals within that confidence category who reported that activity. For example, in the row for "Familiarity with basic research study design" and the column “Prior poster, n (%)”, the value "39 (70.9)" indicates that 39 respondents who rated themselves as highly confident in familiarity with basic research study design had completed a prior poster presentation, and those 39 respondents made up 70.9% of all respondents in that confidence group. Statistically significant results of the Cochran-Armitage tests for trend are indicated by p < 0.05. Non-statistically significant trends (p > 0.05) are indicated with “ns” with the associated p-value below.

Variable	Confidence	Prior poster, n (%)	Prior quickshot, n (%)	Prior podium, n (%)	Prior coauthor publication, n (%)	Prior first author publication, n (%)
Familiarity with basic research study design	Low (n = 6)	3 (50)	1 (16.7)	1 (16.7)	3 (50)	2 (33.3)
	Medium (n = 14)	11 (78.6)	3 (21.4)	7 (50)	7 (50)	5 (35.7)
	High (n = 55)	39 (70.9)	15 (27.3)	24 (43.6)	32 (58.2)	15 (27.3)
	p-value	ns (0.578)	ns (0.501)	ns (0.435)	ns (0.562)	ns (0.578)
Designing and executing research from start to finish	Low (n = 21)	10 (47.6)	2 (9.5)	5 (23.8)	8 (38.1)	2 (9.5)
	Medium (n = 18)	13 (72.2)	5 (27.8)	7 (38.9)	9 (50)	6 (33.3)
	High (n = 36)	30 (83.3)	12 (33.3)	20 (55.6)	25 (69.4)	14 (38.9)
	p-value	0.005	ns (0.054)	0.019	0.019	0.024

**Table 3:** Associations between prior research activities and self-perceived research execution among osteopathic medical students interested in surgery Numbers in each cell represent the count of respondents (n) within a given confidence category who reported the corresponding prior research activity. Percentages in parentheses reflect the proportion of individuals within that confidence category who reported that activity. Statistically significant results of the Cochran-Armitage tests for trend are indicated by p < 0.05. Non-statistically significant trends (p > 0.05) are indicated with “ns” with the associated p-value below.

Variable	Confidence	Prior poster, n (%)	Prior quickshot, n (%)	Prior podium, n (%)	Prior coauthor publication, n (%)	Prior first author publication, n (%)
Conducting comprehensive literature reviews	Low (n = 16)	9 (56.2)	2 (12.5)	4 (25)	7 (43.8)	4 (25)
	Medium (n = 14)	9 (64.3)	3 (21.4)	7 (50)	6 (42.9)	2 (14.3)
	High (n = 45)	35 (77.8)	14 (31.1)	21 (46.7)	29 (64.4)	16 (35.6)
	p-value	ns (0.089)	ns (0.132)	ns (0.188)	ns (0.102)	ns (0.280)
Preparing a manuscript	Low (n = 14)	4 (28.6)	0 (0)	1 (7.1)	5 (35.7)	1 (7.1)
	Medium (n = 20)	11 (55)	2 (10)	6 (30)	8 (40)	4 (20)
	High (n = 41)	38 (92.6)	17 (41.5)	25 (60.9)	29 (70.7)	17 (41.5)
	p-value	<0.001	<0.001	<0.001	0.008	0.009

**Table 4:** Associations between prior research activities and self-perceived statistical skills among osteopathic medical students interested in surgery Numbers in each cell represent the count of respondents (n) within a given confidence category who reported the corresponding prior research activity. Percentages in parentheses reflect the proportion of individuals within that confidence category who reported that activity. Statistically significant results of the Cochran-Armitage tests for trend are indicated by p < 0.05. Non-statistically significant trends (p > 0.05) are indicated with “ns” with the associated p-value below.

Variable	Confidence	Prior poster, n (%)	Prior quickshot, n (%)	Prior podium, n (%)	Prior coauthor publication, n (%)	Prior first author publication, n (%)
Using data analysis tools/software	Low (n = 30)	20 (66.7)	5 (16.7)	13 (43.3)	12 (40)	7 (23.3)
	Medium (n = 26)	17 (65.4)	7 (26.9)	8 (30.8)	18 (69.2)	7 (26.9)
	High (n = 19)	16 (84.2)	7 (36.8)	11 (57.9)	12 (63.1)	8 (42.1)
	p-value	ns (0.232)	ns (0.112)	ns (0.432)	ns (0.073)	ns (0.180)
Statistical skills	Low (n = 37)	23 (62.2)	8 (21.6)	15 (40.5)	17 (45.9)	9 (24.3)
	Medium (n = 23)	18 (78.3)	5 (21.7)	10 (43.5)	17 (73.9)	8 (34.8)
	High (n = 15)	12 (80)	6 (40)	7 (46.7)	8 (53.3)	5 (33.3)
	p-value	ns (0.142)	ns (0.227)	ns (0.680)	ns (0.325)	ns (0.428)

**Table 5:** Associations between prior research activities and self-perceived research dissemination skills among osteopathic medical students interested in surgery Numbers in each cell represent the count of respondents (n) within a given confidence category who reported the corresponding prior research activity. Percentages in parentheses reflect the proportion of individuals within that confidence category who reported that activity. Statistically significant results of the Cochran-Armitage tests for trend are indicated by p < 0.05. Non-statistically significant trends (p > 0.05) are indicated with “ns” with the associated p-value below.

Variable	Confidence	Prior poster, n (%)	Prior quickshot, n (%)	Prior podium, n (%)	Prior coauthor publication, n (%)	Prior first author publication, n (%)
Presenting research through oral and poster presentations	Low (n = 12)	3 (25)	0 (0)	1 (8.3)	5 (41.7)	1 (8.3)
	Medium (n = 9)	6 (66.7)	3 (33.3)	3 (33.3)	3 (33.3)	1 (11.1)
	High (n = 54)	44 (81.5)	16 (29.6)	28 (51.9)	34 (63)	20 (37)
	p-value	<0.001	ns (0.060)	0.005	ns (0.092)	0.025
Ability to teach other medical students basic research fundamentals	Low (n = 24)	11 (45.8)	2 (8.3)	4 (16.7)	8 (33.3)	1 (4.2)
	Medium (n = 18)	12 (66.7)	6 (33.3)	10 (55.6)	13 (72.2)	7 (38.9)
	High (n = 33)	30 (90.9)	11 (33.3)	18 (54.5)	21 (63.6)	14 (42.4)
	p-value	<0.001	0.04	0.006	0.033	0.003

## Discussion

Findings from this national survey reinforce existing literature suggesting that many osteopathic medical students face difficulties translating their research interests into completed projects, presentations, or publications. Nationally, research participation among medical students has increased over the past decade or more. Between 2011 and 2020, the proportion of students involved in faculty-led research rose from 66.3% to 82.5%, and authorship of research papers increased from 40.6% to 55.1% [8]. Despite these promising trends, participation alone does not always yield tangible results. In a 2023 national survey of osteopathic medical students, Ho et al. found that while 85% of respondents reported some form of research experience, only 40% were currently involved in a research project, and just 25% had found their opportunity through their home institution [7]. More than three-quarters of respondents cited barriers such as limited time, lack of access to opportunities, and uncertainty about how to begin. These trends closely align with our findings: although most students in our study reported prior research involvement, 86.7% had never coauthored a peer-reviewed publication, and 79.7% had never published as a first author. This disconnect between interest and output may highlight a persistent gap in structured support, mentorship, and institutional infrastructure, especially for students at schools with fewer built-in research pathways. Without formalized programs that provide guidance, access, and accountability, even highly motivated students may struggle to translate their research engagement into scholarly productivity. While this study focused on students with self-reported research interest, existing literature suggests variability in research training, support, and infrastructure across osteopathic medical schools [9]. For example, some institutions provide structured research programs with dedicated faculty mentorship, while others offer few formalized opportunities. These disparities may influence student confidence and research output, highlighting the need for broader comparative analyses to better understand how institutional factors shape research involvement and output among osteopathic students.

This survey also identified an important disconnect between students’ self-perceived research skills and their research productivity. While most respondents reported high confidence in their familiarity with basic research study design and tasks such as conducting literature reviews and presenting research findings, few had authored peer-reviewed publications. This gap may reflect an overestimation of readiness, as students with minimal research engagement may feel confident in foundational concepts but lack applied experience. Additionally, over half of the respondents reported spending less than two hours per week on research activities, suggesting limited time investment despite high self-reported confidence. These findings imply that while students feel prepared to contribute to research projects, limited access to centralized programs offering structured mentorship, skill development, and research opportunities may hinder students from translating interest into research productivity. However, quantity may not be superior to quality. Williamson et al. recently observed similar findings in osteopathic applicants to surgical residencies, noting that while nearly all applicants reported research experiences, additional experiences beyond the median did not significantly improve match success [9]. This underscores that the quality and structure of mentorship, rather than the quantity of projects, may play a more influential role in advancing both research output and residency competitiveness.

The aforementioned survey by Ho et al. also reported that 62% of students lacked a research mentor, and only 13% had a research mentor affiliated with their medical school [7]. The lack of mentorship may be partially explained by findings from a review of osteopathic medical school research programs by Hamby et al., which reported that most schools hosting student research programs required students to independently secure faculty mentorship for their projects [10]. Given that mentorship is linked to increased research productivity and enhanced career planning among medical students [11], addressing this gap remains essential.

Our survey also revealed that osteopathic medical students reported the lowest confidence in statistical analysis and the use of data analysis tools or software. These gaps are consistent with a global mixed-methods systematic review and meta-analysis conducted by Amgad et al., which found that medical student participation in research was often limited to tasks such as literature review and data collection, with fewer students involved in research methodology design or statistical analysis [11]. Students who participate in structured research curricula or mentored programs have demonstrated higher statistical literacy [12]. Conversely, while all osteopathic medical schools include basic biostatistics education as part of the core curriculum, limited opportunities for applied training, such as using statistical programs or conducting independent analyses, may contribute to skill deficiencies in this domain. These findings are also supported by students’ self-reported familiarity with specific study types, such as case reports, which typically do not require the utilization of research methodology design and statistical analysis to the same extent as clinical trials or meta-analyses may. While statistical skills represent one of the weaker domains in this survey, some students may gain additional skills through active dissemination of their research.

Research dissemination, such as through poster or podium presentations, may serve as an avenue to improve research skills and confidence. Findings from our survey suggest that students who had presented their research reported significantly greater confidence in manuscript preparation and in teaching research skills to peers. Presenting data often requires students to interpret and articulate statistical findings, fostering a more applied understanding of analysis methods. This highlights research dissemination as a potential method for advancing research competencies. Consistent with our findings, prior evaluations of structured research programs that emphasize poster and abstract presentations have demonstrated that active engagement in research communication fosters skills such as critical thinking, data interpretation, and scientific writing [13]. Encouraging medical students to participate in research presentations may be a practical intervention to improve research skills among osteopathic medical students.

These challenges are not unique to osteopathic medical students. Students pursuing smaller, more competitive specialties also face similar barriers. For instance, a national survey of U.S. medical students pursuing cardiothoracic surgery found that 57% lacked a home training program in that specialty and 72% had no cardiothoracic interest group. Mentorship and networking were the most highly desired forms of support [14]. To address this gap, the Society of Thoracic Surgeons partnered with the Thoracic Surgery Medical Student Association to launch a national mentorship initiative, connecting medical students with cardiothoracic surgery residents and faculty mentors across institutions [15]. Similarly, recent efforts have attempted to bridge structural gaps in research access among osteopathic medical students. One such initiative, the Osteopathic Research Initiative for Growth and INnovation in Surgery (ORIGINS), connects osteopathic medical students with research mentors to complete projects over the course of an academic year. Another example is REAM ORTHO, which matches osteopathic medical students interested in orthopedic surgery with faculty mentors, collaborative research projects, and publishing opportunities [16]. Now supported by national organizations, these models highlight how coordinated mentorship, accountability, and longitudinal engagement can help foster scholarly productivity in under-resourced settings. However, isolated programs alone cannot overcome the larger institutional and systemic barriers that limit research access. Broader, scalable initiatives are needed to ensure that all students have equitable opportunities for meaningful research participation. National, collaborative programs that expand access to mentorship, statistical training, and structured research guidance may help convert students’ motivation into tangible research presentations and publications, even at institutions with limited resources. At the same time, these interpretations should be viewed with appropriate caution, given the cross-sectional study design. The associations identified in this survey cannot establish causal relationships, and the findings should therefore be considered exploratory. While students with prior dissemination experience reported higher confidence in several research skills, the survey cannot determine whether these activities increased confidence or whether more confident students were simply more likely to pursue dissemination opportunities. Future longitudinal studies are needed to determine whether changes in confidence translate into measurable improvements in research productivity, residency preparation, or long-term scholarly engagement.

Limitations

With only 75 respondents, the generalizability of the findings to the broader osteopathic medical student population may be limited. Selection bias is likely, as students with a stronger interest in research or surgical careers may have been more inclined to complete the survey. Recruitment through professional organizations and a national surgical conference may have disproportionately attracted students who were already more research motivated or professionally engaged, further limiting sample representativeness. Osteopathic students pursuing surgical specialties may place greater emphasis on research compared to peers pursuing primary care, where research involvement may not be as heavily prioritized. Additionally, the cross-sectional design limits the ability to infer causality between research experience and self-perceived competency. Although the survey was distributed nationally, we could not ensure balanced representation across geographic regions or osteopathic institutions. Further selection bias may have resulted from voluntary recruitment through social media and conference-based outreach, which may have attracted respondents already more engaged in research or student organizations. Finally, without longitudinal follow-up, we could not assess whether early research engagement or self-confidence in skills leads to long-term scholarly output or improved residency preparation.

## Conclusions

Osteopathic medical students pursuing surgical specialties appear motivated and confident in basic research skills, yet encounter systemic barriers that limit their research output. Enhancing structured research programs, mentorship opportunities, and skill development initiatives, particularly through national collaborations, will be crucial in bridging the gap between student interest and scholarly productivity. By addressing identified deficiencies in mentorship and training, the osteopathic medical education community may be able to support students in contributing meaningfully to research and in strengthening their residency applications in competitive surgical fields.

## Appendices

Appendix A: Research Skills and Goals Assessment Survey

1. Have you completed this survey previously? a. Yes b. No

2. Age a. 18-25 b. 26-30 c. 31-40 d. 41+

3. Gender a. Cisgender male b. Cisgender female c. Transgender male d. Transgender female e. Other (please specify)

4. Race (Select all that apply)a. American Indian or Alaska Native b. Asian c. Black or African American d. Native Hawaiian or Other Pacific Islander e. White f. Other (please specify)

5. Military Service a. Yes b. No

6. Socioeconomic Status During K-12 Education a. Below poverty line (family income < federal poverty threshold) b. Low income (family income < 2x federal poverty threshold) c. Middle income (family income 2-4x federal poverty threshold) d. High income (family income > 4x federal poverty threshold)

7. Type of Medical School a. University/academic b. Community/community-affiliated c. Mix of academic and community

8. Geographic Location of Medical School/Medical School Location: State/Country (if outside the US) a. Pacific b. Mountain c. West North Central d. East North Central e. West South Central f. East South Central g. Middle Atlantic h. South Atlantic i. New England

9. Does your school have a home surgery program? a. Yes b. No

10. Have you participated in any previous research projects? a. Yes b. No

11. Are you currently involved in any ongoing research projects? a. Yes b. No

12. Previous Research Presentations: (Select all that apply) a. Oral Podium Presentation (0, 1, 2, 3, 4+) b. Oral Quickshot (0, 1, 2, 3, 4+) c. Poster Presentation (0, 1, 2, 3, 4+)

13. Previously Published Research: (Select all that apply) a. Yes, as first author (0, 1, 2, 3, 4+) b. Yes, as co-author (0, 1, 2, 3, 4+) c. None

14. Intended Specialty a. General Surgery b. Neurosurgery c. Orthopedic Surgery d. Urology e. Plastic Surgery f. ENT/Otolaryngology g. Ophthalmology h. Undecided

15. Hours per Week Currently Dedicated to Research a. 0-2 hours b. 3-5 hours c. 6-10 hours d. 11-15 hours e. 16+ hours

16. I have a clear idea of what my long-term research focus is. Strongly disagree 1 2 3 4 5 Strongly agree

17. I am confident in my ability to use research databases (e.g., PubMed, Google Scholar, etc.) to find relevant articles. Strongly disagree 1 2 3 4 5 Strongly agree

18. I am confident in my ability to critically evaluate the quality and relevance of research studies. Strongly disagree 1 2 3 4 5 Strongly agree

19. Which research methodologies are you already familiar with? (Select all that apply) a. Systematic Reviews b. Meta-analyses c. Case Reports d. Surveys e. Clinical Trials f. Basic Science/Bench Research

20. I am familiar with basic research study designs (e.g., observational, randomized controlled trials, cohort studies, etc.) Strongly disagree 1 2 3 4 5 Strongly agree

21. I am confident in my own ability to conduct comprehensive literature reviews. Strongly disagree 1 2 3 4 5 Strongly agree

22. I am proficient in using data analysis tools/software (e.g., SPSS, Excel, R). Strongly disagree 1 2 3 4 5 Strongly agree

23. I am comfortable preparing a manuscript, including identifying focus, writing sections, and communicating with editors. Strongly disagree 1 2 3 4 5 Strongly agree

24. I am confident in presenting research findings through oral and poster presentations. Strongly disagree 1 2 3 4 5 Strongly agree

25. I feel confident in my statistical skills. Strongly disagree 1 2 3 4 5 Strongly agree

26. I feel that I have the skills to design and execute a research project from start to finish. Strongly disagree 1 2 3 4 5 Strongly agree

27. I am confident in my ability to teach other medical students basic research fundamentals (e.g., how to form a research question; how to start a project; how to search and develop a literature review, etc). Strongly disagree 1 2 3 4 5 Strongly agree
